# Books Received

**Published:** 1869-03-15

**Authors:** 


### BOOKS RECEIVED.

PATHOLOGICAL PHENOMENA GENERALIZED, by H. BACKUS, Montevallo, Alabama.

The above is the title to a modest looking pamphlet of about sixty pages, in which the author attempts to reduce by induction, all pathological phenomena to the operation of one general law, and deduce them all from one common cause—congestion. The introduction, which comprises about one-fifth of the entire pamphlet, bears the evidence of an extensive acquaintance with the works of the later English metaphysical writers; and indeed, at its conclusion, the author very gracefully acknowledges his indebtedness to Lewis, Mill, and Combe; to the latter, however, he owes but little, and to none of them much, as the pupil is more logical than his masters—the imitator than the models. He modestly intimates an objection which has been urged, that he "an obscure and unlearned man, should hope to succeed with a subject where so many mighty intellects have failed." He "trusts that some of our readers will reserve this point until we have gone through with what we have to say, remembering that a 'cripple in the right way will beat a racer in the wrong.'"

As the author is unknown to us, we are not prepared to dispute his claims to the honors of obscurity, for in these days when notoriety is so frequently attained dishonorably, obscurity may not improbably be a criterion of merit. But unlearned he is not. Of this popular characteristic we would deprive him most decidedly, were it not for the fact that he has destroyed the validity of his title thereto by the display of a most extensive and comprehensive acquaintance with standard authors, both American and European, professional, and (apparently) philosophical. The author claims, that as all physical phenomena after having been successfully referred to Supernatural causes, Metaphysical Entities, and finally through the inductive method of Positive Science—the law of gravitation. So also, Pathological Phenomena having passed through identical phases, must eventually be reduced by the same method to the same cause. He claims farther, and justly too, for his method the advantage, that it proceeds "with cultivated caution from the known to the unknown." He asserts that as we know nothing of vital forces we can predict nothing therefrom; "we cannot reach the unknown through the unknown." He claims for Pressure, which is resolvable into Gravitation, a predominant agency in the production of pathological phenomena, and refers to this cause both Hypertrophy and Dilatation of the heart, which, in their turn, become agents for propagation of the same condition in other organs.

While the author brings to the support of his theory, illustrations drawn from the effects of congestion upon the nervous system, more especially upon its great centres, he is silent as to the influence of the nervous system upon the circulatory in inducing or removing this condition. Having followed him step by step, and believing his propositions well framed and well sustained too, up to the limit of his conclusions, we must at this point express the opinion that he has stopped short of the ultimate fact to which a sufficiently broad generalization of pathological phenomena might have led him. He appears throughout his argument to isolate his field of observation and experiment too completely from its external relations, while asserting the desire to reduce pathological phenomena to the operation of physical forces he takes, we think, too partial and limited a view of their correlations. If his conclusion be admitted, which it must be, that the ultimate factor in *his* pathological induction, be congestion, the question arises, have we yet reached the utmost limit to which induction can lead us? If not, then comes the further question, what produces congestion? A link must here be supplied to connect this condition with external physical phenomena, and we apprehend that in the modification of the nervous system, both functional and structural, as induced by external agencies, physical or psychical, and reacting upon the circulatory system to facilitate or oppose congestion, his ultimate factor will be found the missing link, which shall prove the last one in the chain of phenomena extending from the operations of external influences to those complex pathological problems which puzzle the best of us so sadly.

We have read this essay with much pleasure and gratification, and commend it as the work of one who not satisfied to know things as they seem, desires that higher knowledge, the "*rerum cognitio per ultimas altissimasque rationes.*"* The essay, brief as it is, deserves a more extended notice than it is possible to accord to it within the narrow limits at our command, but we feel confident that to those who appreciate logical writing it will prove a benefit, and to those who do not, a benefit yet greater, as their need is greater.

WE HAVE RECEIVED a copy of the Constitution and By-Laws of the Gynæcological Society, of Boston, an organization having for its object the study and advancement of the science whose name it bears. It is the intention of the founders to establish, by the appropriation of two-thirds of its receipts

---

* *Salvator Tongiorgi, Institutiones Philosophicæ. Romæ, 1862.*

thereto, a library of works, bearing upon their own specialty. Its officers are: Winslow Lewis, President; Horatio R. Storer, Secretary; George H. Bixley, Treasurer. We are glad to see this tendency to concentration of thought and effort upon the cultivation of special departments of medical science as manifested in the organization of this and kindred societies. It is especially through such agencies that sciences are to be advanced. We wish the Gynæcological Society of Boston all the success that the names of its founders assure for it.

Dr. J. M. Bigelow, of Detroit, sends us a treatise upon Svapnia, or purified Opium, for which he claims certain advantages over the crude drug or any of its ordinary preparations; such as uniformity of composition, and hence of therapeutic effect and freedom from some of the irritant effect upon the nervous system so frequently consecutive to the use of opium, and in many cases entirely contra-indicating the use of this "great boon to humanity." It is very desirable that the less commonly known alkaloids of this drug—Codeia, Thebaia and Narceia, should be made more accessible to the practioner than at present. The known therapeutic action of the latter principles especially rendering it a most desirable agent in the treatment of a large class of pathological conditions of the nervous centres to which the other alkaloidsand the crude drug itself are entirely inapplicable.

ON THE IDENTITY OF THE WHITE CORPUSCLES OF THE BLOOD WITH THE SALIVARY PUS AND MUCOUS CORPUSCLES. By Joseph G. Richardson, M. D., formerly Resident Physician at the Pennsylvania Hospital.

Dr. Richardson, already quite well known to the profession as a Micrograph, presents in this paper his conclusions, summarized in the title, from observations made with a 1-25 objective (of Wales) upon these corpuscles and corroborating the conclusions of Cohnheim (first published in America in The Journal of Oct. 15, 1868), regarding the amaboid movements of the white corpuscles of the blood and their mode of exit through the walls of the vessels, claims priority in the discovery of their identity. The subject is full of interest, embracing a wide field for scientific investigation, and promising important data for practical deduction, and if the author's conclusions be not absolutely correct, they are, to say the least, deserving of critical examination by microscopic and physiological experts. It is by the aid of the microscope exercised in the field of histology, and by organic chemistry, that we must hope to unravel the more intricate physiological processes, without a correct appreciation of which we must be content to read the signs of disease as "through a glass darkly."

A HISTORY OF THE MEDICAL DEPARTMENT OF THE UNIVERSITY OF PENNSYLVANIA, from its foundation in 1765, with Sketches of the Lives of Deceased Professors. By Joseph Carson, M. D., Prof. of Materia Medica and Pharmacy in the University of Pa.; Member of the American Philosophical Society, etc. Price $3.00. *Lindsay & Blakiston.* 1869. Philadelphia.

PENNSYLVANIA HOSPITAL REPORTS, Vol. 2., 1869. Price $5.00. *Lindsay & Blakiston.* Philadelphia.

TREATISE ON THE DISEASES OF THE EAR, including the Anatomy of the organ. By ANTON VON TROLSCH, M. D., Prof. in the University of Wurzburg, Bavaria. Translated and edited by D. B. ST. JOHN ROOSA, M. A., M. D., Clinical Prof. of the Diseases of the Eye and Ear, in the University of New York, Surgeon to the Brooklyn Eye and Ear Hospital, formerly Surgeon to the New York Eye and Ear infirmary, etc. Second American from the Fourth German edition. Price $4.50. *Wm. Wood & Co.*, New York. 1869.

A TREATISE ON THE DISEASES OF INFANCY AND CHILDHOOD. By J. LEWIS SMITH, M. D., Curator to the Nursery and Child's Hospital, New York, Physician to the Infants' Hospital, Ward's Island, Professor in Bellevue Hospital Medical College, New York.

SYPHILIS AND LOCAL CONTAGIOUS DISORDERS. By BERKELEY HILL, M. D. London, F. R. C. S., Assistant Surgeon to University College Hospital; Teacher of the Use of Surgical Apparatus in University College, and Surgeon to Out Patients at the Lock Hospitals. *Henry C. Lea.* Philadelphia. 1869.

THE PART TAKEN BY NATURE AND TIME IN THE CURE OF DISEASES. A Dissertation for which a Prize was awarded to JAMES F. HIBBERD, M. D., by the Massachusetts Medical Society. 1868. Boston: *David Clapp & Son*, 334 Washington St. From the author.

